# Enterovirus 71 targets the cardiopulmonary system in a robust oral infection mouse model

**DOI:** 10.1038/s41598-019-47455-3

**Published:** 2019-07-31

**Authors:** Chih-Shin Chang, Chun-Che Liao, An-Ting Liou, Ya-Shu Chang, Ya-Ting Chang, Bing-Hsiean Tzeng, Chien-Chang Chen, Chiaho Shih

**Affiliations:** 1 Program in Molecular Medicine, National Yang-Ming University and Academia Sinica, Taipei, Taiwan; 2 Institute of Biomedical Sciences, Academia Sinica, Taipei, Taiwan; 3 Institute of Microbiology and Immunology, National Yang-Ming University, Taipei, Taiwan; 4 Taiwan International Graduate Program in Molecular Medicine, National Yang-Ming University and Academia Sinica, Taipei, Taiwan; 5 Cardiovascular Section, Far Eastern Memorial Hospital and Tri-Service General Hospital, National Defense Medical Center, Taipei, Taiwan

**Keywords:** Viral pathogenesis, Infection

## Abstract

Severe infection with the re-emerging enterovirus 71 (EV71 or EV-A71) can cause cardiopulmonary failure. However, in patients’ heart and lung, viral protein has not been detected. In mouse models, heart disease has not been reported. EV71-infected brainstem is generally believed to be responsible for the cardiopulmonary collapse. One major limitation in EV71 research is the lack of an efficient oral infection system using non-mouse-adapted clinical isolates. In a robust oral infection NOD/SCID mouse model, we detected EV71 protein at multiple organs, including heart and lung, in 100% of moribund mice with limb paralysis. Infiltrating leukocytes were always detected in heart and muscle, and VP1-positive M2 macrophages were abundant in the lung. Functional dissection on the pathogenesis mechanism revealed severe apoptosis, inflammatory cytokines, and abnormal electrocardiogram (EKG) in orally infected hearts. Therefore, cardiopulmonary disease could be one plausible cause of death in this mouse model. Inoculation of EV71 through an oral route resulted in viral infection in the intestine, viremia, and EV71 appeared to spread to peripheral tissues via blood circulation. Infectious virus was no longer detected in the blood on day 5 post-infection by the plaque formation assay. We demonstrated that both EV71 clinical isolate and cloned virus can target the cardiopulmonary system via a natural infection-like oral route.

## Introduction

Epidemics of enterovirus 71 (EV71 or EV-A71) occurred frequently worldwide^1–4^. In a recent outbreak in Shanghai, China, near 1000 deaths of children were reported^5^. Infection with EV71 in children is associated with a wide range of severity, including hand-foot-and-mouth disease (HFMD), encephalitis, acute flaccid paralysis, tachycardia (135–250 heartbeats per minute), cardiopulmonary failure, and death. EV71 is closely related to poliovirus, hepatitis A, and coxsackievirus^6–9^. Recently, another close relative of EV71, enterovirus D68 (EV-D68), is emerging as a global infectious disease, particularly in North America^10^. In the 2014 outbreak, at least 2000 cases of EV-D68 were reported from 20 countries worldwide. EV-D68 is also associated with severe respiratory disease and acute flaccid paralysis. Altogether, reemerging non-polio enterovirus represents a new major threat to the public health. While formalin-inactivated EV71 vaccine will be clinically available^11,12^, current treatment for enterovirus remains supportive, and no FDA-approved therapeutics for EV71 is currently available on the market.

**Figure 1:**
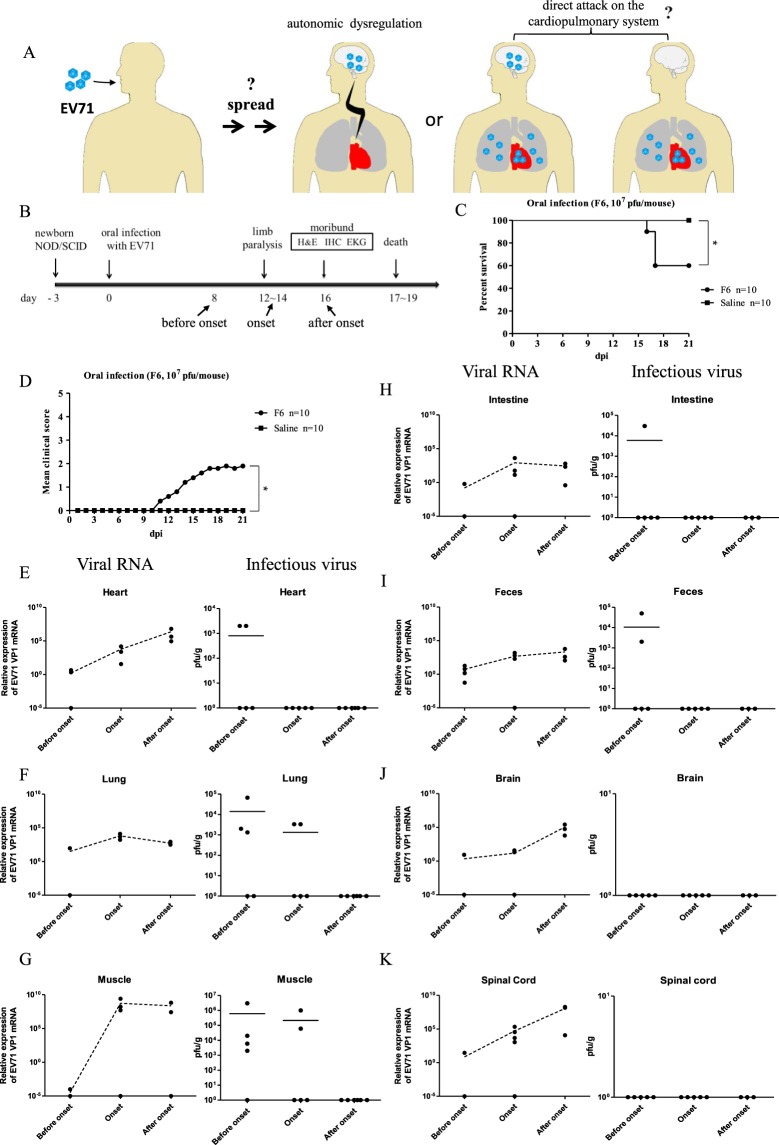
Infection kinetics of EV71 RNA and plaque forming activity in multiple organs in orally infected NOD/SCID mice. (**A**) Cartoon illustration of the pathogenesis mechanisms of EV71 oral infection. Left panel: A conventional hypothesis postulates that EV71 can cause tachycardia and lung edema from the dysregulated autonomic nerve system in the infected brain. Right panel: An alternative hypothesis is to postulate that EV71 can directly attack heart and lung with or without brain infection. It remains unclear how EV71 can spread to the peripheral tissues after oral intake. (**B**) Three-day-old NOD/SCID mice were infected orally with 10^7^ pfu of purified EV71-F6 (from infectious clone in Fig. S1) or the saline control. Infected mice with limb paralysis were sacrificed on day 16 post-infection. (**C**,**D**) Survival curve and clinical score in NOD/SCID mice orally inoculated with a cloned virus EV71-F6. (**C**) In a time course experiment, death was observed around day 16, and survival rate is around 60%. Each experimental group contained 10 mice. (**D**) Clinical scores were defined as follows: 0, healthy; 1, wasting, or ruffled hair; 2, limb weakness; 3, paralysis in only 1 limb; 4, paralysis in 2 to 4 limbs; 5, death. Each experimental group contained 10 mice. *p value less than 0.05. (**E–K**) Kinetic profiles of viral RNA (qPCR) and infectivity (pfu) from 6 different tissues and feces were compared before, during, and after disease onsets. No apparent clearance of replicating viral RNA was noted. Each solid dot indicates one individual mouse. The dotted lines represent an average of several independent experiments. Horizontal bars in the right column of infectious virus (E-K) indicate averages of pfu.

Sudden death and cardiopulmonary collapse are most common in fatal cases of EV71 infection. It is generally believed that mortality is due to neurogenic pulmonary edema and cardiac decompensation from CNS infection, inflammation, and the consequent sympathetic hyperactivity^1–3,13^ (Fig. 1A, middle). Although there are data for encephalomyelitis, no experimental evidence for viral myocarditis^14–17^. MRI studies on cardiopulmonary failure cases showed hyperintensity of the posterior aspect of the medulla^14^. In addition, left ventricular ejection fractions (LVEF) on cardiac echography of patients were found to be lower than the average of normal children. Pulmonary edema of fulminant EV71 infection was found to be very well correlated with left ventricular dysfunction^18,19^. One critical issue here for human patients is whether heart and lung can be directly attacked by EV71 (Fig. 1A, right panel), or whether the sole cause of cardiopulmonary failure is the sympathetic hyperactivity from the CNS infection (Fig. 1A, middle).

Previously, oral infection with mouse-adapted strains of EV71 has been tested in various mouse models. However, mouse-adapted strains accumulated many artificial mutations during serial passages through the mouse brain^20^. Therefore, they no longer can faithfully represent the parental clinical isolates from patients. In the model of hSCARB2 transgenic mice, a Japanese Isehara strain of EV71 was used for oral infection^21^. However, the oral infection efficiency in this model was low (2/50). Oral infection with clinical isolates of EV71 (10^6^–10^7^ pfu/mouse) was reported at a moderate efficiency around 15–40% in immunodeficient AG129 mice^22^. Recently, a robust oral infection system was established in the NOD/SCID mouse model. Approximately 71% of orally infected mice developed paralysis and death^23^. To date, efficient oral infection with EV71 clinical isolates (non-mouse-adapted) can be conducted only in the immunodeficient NOD/SCID mouse model.

In this study, by using the more efficient oral infection system of NOD/SCID mice, we detected EV71 specific protein VP1, viral RNA, and infectivity by plaque forming assay in multiple organs, with near 100% frequencies of VP1 and viral RNA in heart and lung of moribund mice. Massive infiltration of leukocytes can be detected in both tissues. Abundant VP1-positive macrophage (M2 type) and pneumocytes (type I and type II) were also observed in the infected lung tissue. Using an intraperitoneal (i.p.) route in the 3-day old NOD/SCID mice, we also identified viral VP1 protein in both heart and lung. Infected cardiomyocytes lost the normal pattern of connexin 43 (a gap-junction channel protein). Intriguingly, functional electrocardiogram (EKG) revealed abnormal electrophysiology in 70–100% of orally infected moribund mice, but not in i.p.-inoculated moribund mice. Furthermore, severe apoptosis was also detected in 70% of cardiomyocytes of orally infected moribund mice, and only less than 20% in i.p. infected moribund mice. Except for one mouse, we have so far detected no VP1 protein in the brain and spinal cord. In our oral infection model, there was no apparent difference in the cardiopulmonary pathogenesis between cloned virus F6 and uncloned clinical isolate F23 (quasispecies), despite their different degrees of sequence divergence (Materials and Methods). Our results demonstrated, for the first time, that the cardiopulmonary failure could originate directly from EV71 infection, inflammation and pathogenesis in the heart and lung in this oral infection model. The potential mechanisms of cardiomyocyte apoptosis and the route of virus spreading from gut to heart and lung are discussed.

## Results

### Infection kinetics of EV71 in multiple organs in orally infected NOD/SCID mice

EV71 is mainly transmitted via an oral-fecal route in natural infection (Fig. 1A). Because cardiopulmonary collapse is a fatal cause in severe cases of EV71 infected children, we examined the tissue distribution of EV71 in an oral infection mouse model (Fig. 1B). Three-day-old NOD/SCID mice were inoculated orally with cloned EV71-F6 virus at 10^7^ pfu/mouse. This cDNA clone from clinical isolate of EV71-F6 has been shown to be infectious *in vitro* by RD cells and *in vivo* by i.p. injection (Fig. S1). In general, approximately 50% of the orally infected NOD/SCID mice developed limb paralysis on 12–14 dpi, moribund status on 16 dpi, and death on 17–19 dpi (Fig. 1C,D). A survival curve and the clinical score are summarized in Fig. S2. In a time course experiment (before, during, and after disease onsets), we examined the infection kinetics of EV71 in the cardiopulmonary system (heart and lung), CNS (brain and spinal cord), GI tract (intestine and feces), and paralyzed limb muscle. EV71 specific RNA and plaque forming activity were extracted from dissected tissues (Materials and Methods), and measured by qPCR and *in vitro* infection of RD cells, respectively (Fig. 1E–K). In general, clearance of viral infectivity appeared to occur much earlier than the clearance of viral RNA. For example, except for the CNS, plaque forming activity were detected in all tissues examined before disease onset (before 12 dpi), and remained detectable only in lung and muscle at the stage of disease onset (around 12–14 dpi), but undetectable in all tissues after onset (after 16 dpi). In contrast, we observed no significant decrease or clearance of viral RNA in all tissues at all stages before sacrifice of the infected mice.

### Tissue distribution of VP1 protein in orally infected mice

**Figure 2:**
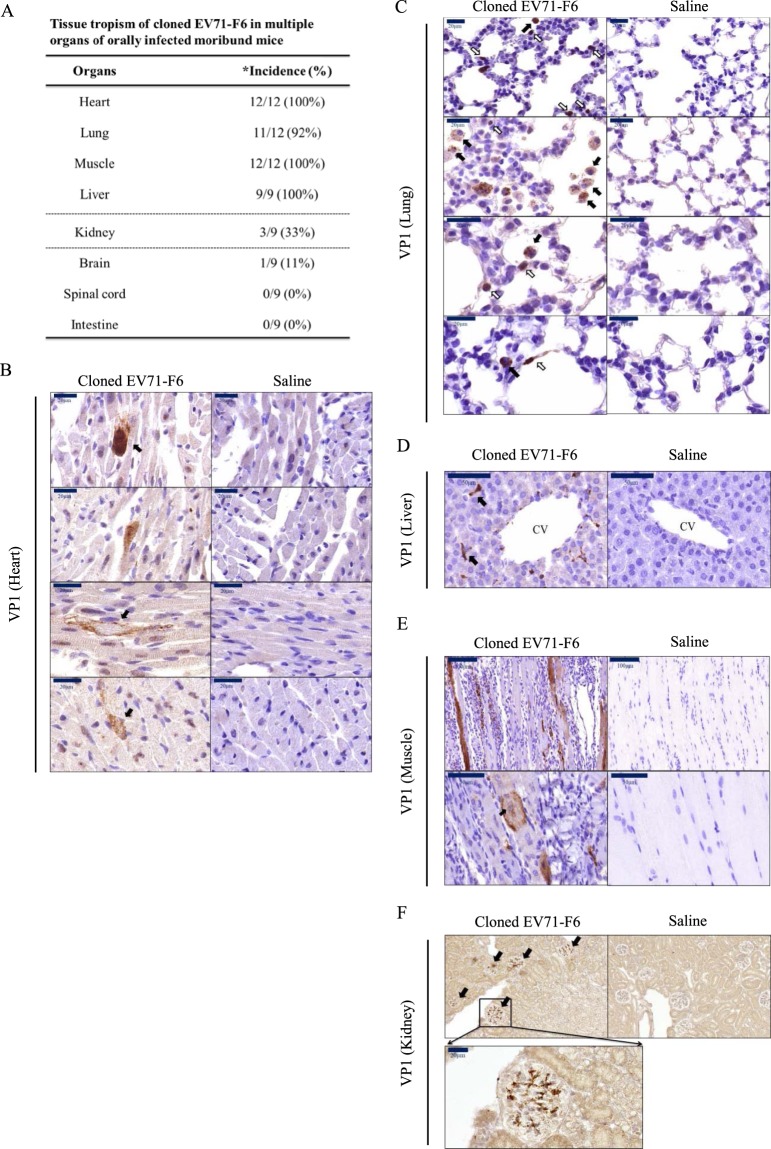
Tissue distribution of EV71 in orally infected NOD/SCID mice. Experimental design was as described in Fig. 1B. Paraffin-embedded sections of different tissues from moribund mice were visualized with immunohistochemistry using a rabbit antibody specific for EV71 protein VP1. (**A**) A summary of EV71 tissue tropism in this oral infection model. * incidence rates represent the numbers of VP1-positive organs divided by the total number of examined moribund mice. Strong signals of VP1 can be detected in (**B**) cardiomyocytes, (**C**) alveoli (black arrow) and epithelial-like cells (white arrow) in the lung, (**D**) morphologically characteristic Kupffer cells around the central vein, (**E**) myocytes of muscle. Centralized nuclei (black arrow) is known to be associated with muscle regeneration, (**F**) kidney glomerulus.

In addition to the viral RNA and pfu activity, we examined viral protein VP1 by immunohistochemistry (IHC) staining, including heart, lung, muscle, and liver (Fig. 2A). Multiple organs from the moribund mice were found positive at near 100% incidence. In contrast, except for one case in the brain (1/9), no VP1 was ever detected in the CNS and intestine. Kidney showed an intermediate incidence rate for VP1 (33%). Strong signals of VP1 can be identified in cardiomyocytes (Fig. 2B, upper panel), and loss of the normal morphology or the muscle-characteristic striated pattern was observed in some heart sections (Fig. 2B, lower panel). In the lung sections (Fig. 2C), VP1-positive signals can be identified in the center of alveoli (black arrow) and in lung epithelial cells (white arrow). VP1 signals were also detected in kupffer-like cells near central vein (CV) in the liver (Fig. 2D). Hindlimb muscle sections revealed VP1-positive muscle bundles, massive leukocyte infiltration, and histopathological changes in the muscle morphology (Fig. 2E). A swollen and distorted muscle cell appeared to have lost the characteristic striated pattern, and its centralized nuclei (solid arrow) indicated muscle regeneration in response to injury. In kidney, VP1 signals were occasionally detected in the glomerulus (Fig. 2F). In only one exception, we detected VP1 signal in cerebellum (Fig. S3).

### Severe apoptosis and pathology in cardiomyocytes

**Figure 3:**
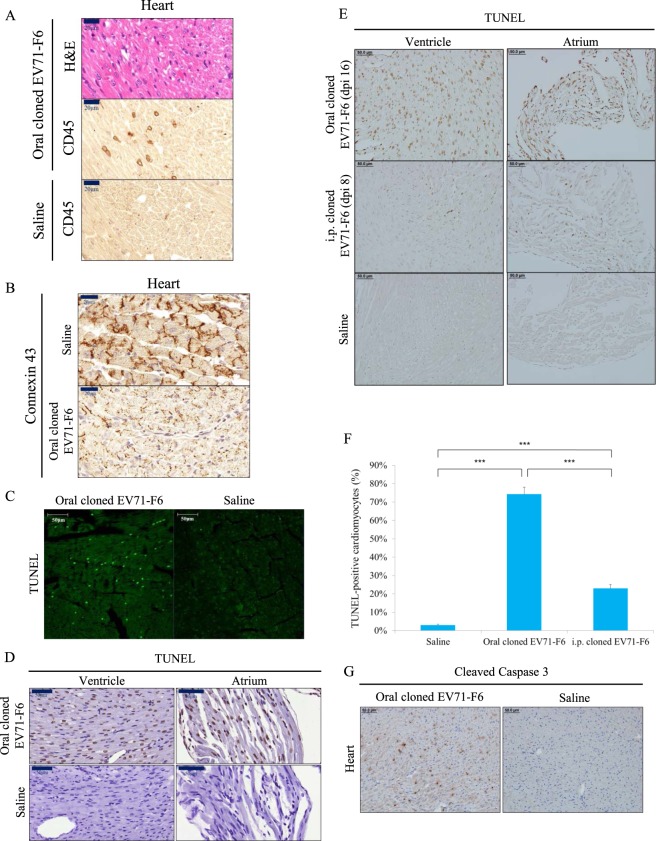
Severe apoptosis and pathology in the orally infected heart. Experimental design is as outlined in Fig. 1B. (**A**) Heart sections from moribund mice were examined with H&E, and immunohistochemistry staining for pan-leukocyte infiltration using an anti-CD45 antibody. (**B**) The gap junction channel protein connexin 43 at the border of cardiomyocytes were lost in EV71-infected mice. (**C**) IFA TUNEL assay detected massive host DNA fragmentation. (**D,E**) Extensive apoptosis in ventricle and atrium was detected by IHC in orally infected (16 dpi), but not in i.p.-infected (8 dpi), moribund mice. See Table 2 for tissue distribution of VP1 in the i.p.-infected group. (**F**) Oral infection resulted in near 70% apoptotic cardiomyocytes by TUNEL assay (Merck). ***p < 0.0001. (**G**) Activated caspase 3 was detected by IHC.

In this oral infection model, EV71 can target at multiple organs (Figs 1 and 2). Because failure of the cardiopulmonary system is responsible for the death of severe EV71 infection, we focused our subsequent studies only on both heart and lung. We first detected CD45-positive leukocytes in all sections of the entire heart (Fig. 3A). Connexin 43 is a gap junction membrane protein present in the intercalated disc on the surface of cardiomyocytes^24^. In animals orally infected with the cloned virus F6, the normal pattern of connexin 43 in the ventricles was partially  lost (Fig. 3B). By immunofluorescence microscopy, the percentage of TUNEL positive (apoptotic) nuclei (less than 30%) in heart sections from orally infected mice was highly increased by orders of magnitude over the saline control (Fig. 3C). Similar results in both ventricle and atrium were obtained, when TUNEL assays were conducted by using IHC kits from two different vendors (Fig. 3D,E). In contrast, very low backgrounds of apoptotic cells were scored quantitatively in the saline control or i.p. infected animals (Fig. 3E,F). The cleaved form of caspase 3 is a well-known marker of active apoptosis. By IHC, we detected strong signals of cleaved caspase 3 in orally infected heart (Fig. 3G). Taken together, severe apoptosis in cardiomyocytes was observed frequently in orally infected heart.

### Abnormal electrocardiogram (EKG)

**Figure 4:**
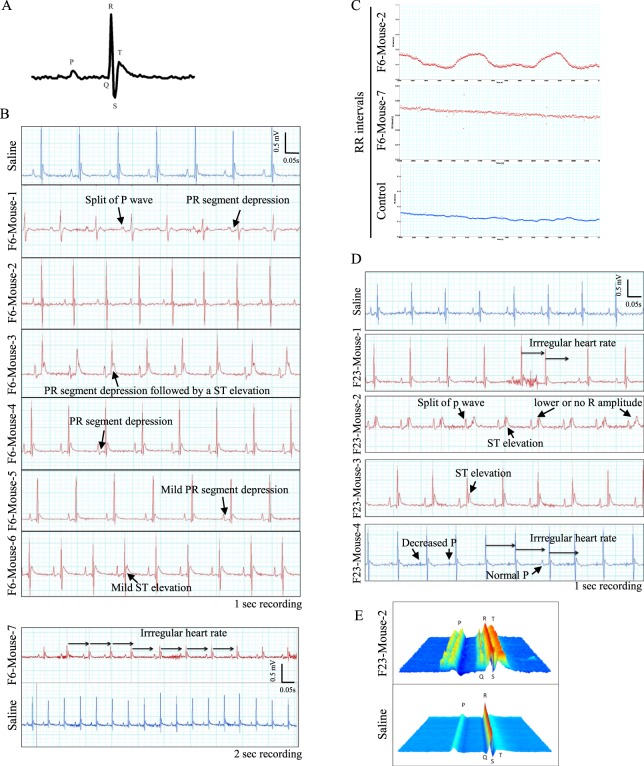
Functional abnormality in electrocardiogram (EKG) in an oral infection mouse model. The 3-day-old NOD/SCID mice were infected orally with 10^7^ pfu of EV71-F6 (n = 7), EV71-F23 (n = 4) or normal saline. Infected mice were examined by EKG analysis on day 16 post-infection. (**A**) A representative configuration of a typical mouse electrocardiogram is derived from an average of four consecutive heartbeats of an uninfected littermate. P wave: the electrical manifestation of spread of atrial excitation; QRS complex: the electrical manifestation of spread of ventricular excitation; T wave: repolarization of the ventricles. (**B**) EKG in one-second recording showed a consistent split of P wave in F6-Mouse-1, PR segment depression in F6-Mouse-1, -3, -4 and -5, and ST elevation in F6-Mouse-3, and mild ST elevation in F6-Mouse-6. In two-second recording, irregular heart rate was evident in F6-mouse-7. (**C**) RR intervals showed irregular heart rate in F6-Mouse-2 and -7. (**D**) EKG showed irregular heart rate in mouse F23-Mouse-1 and -4, shortened or no R amplitude in mouse F23-Mouse-2, apparent ST elevation in F23-Mouse-2 and -3, and nearly abolished P wave in mouse F23-Mouse-4. (**E**) A Waterflow Plot resolved split P peaks and R amplitude in F23-mouse-2.

To investigate further the electrophysiology of the infected heart, we compared the EKG between infected and uninfected control mice. As shown in Fig. 4A, a typical EKG is compiled from an average of four consecutive heartbeats of an uninfected (saline control) littermate on 16 dpi. Various abnormal EKG patterns were very common in the orally infected group (70–100%), including the split or decrease of P wave, reduced R amplitude, PR segment depression, ST elevation, and irregular heart rate (Fig. 4B–D; summarized in Table 1). The split of P or R waves can be more clearly resolved, when using a Waterflow Plot in a 5-min recording (Fig. 4E). These EKG phenotypes can be detected at a high frequency in mice orally infected with either the cloned virus (F6) or clinical isolates (F23, quasispecies) (Table 1). However, except for one arrhythmia case in each experimental group (n = 5) i.p.-infected with either a clinical isolate F23 or the cloned virus F6, we observed no abnormal EKG profiles (Table 1 and Fig. S4). In summary, EV71 pathogenesis of orally infected heart includes inflammation, apoptosis and abnormal electrophysiology.

**Table 1:** Comparisons of EKG profiles among different sources of EV71 viruses via different routes of inoculation.

Routes & EV71 viral strains	EKG phenotypes	Incidence (%)	possible causes	references
Oral, F6 (infectious clone)	PR segment depression	4/7 (57%)	acute perimyocarditis	^31,32^
	irregular heart rate	2/7 (29%)	supraventricular event (SVE), sinus arrhythmia	^33^
	ST elevation	2/7 (29%)	myocardial infarction	^34^
	split of P wave	1/7 (14%)	atrial enlargement, dilated cardiomyopathy	^35^
	at least one EKG phenotype	5–7/7 (>70%)		
Oral, F23 (clinical isolate)	irregular heart rate	2/4 (50%)	atrial premature contraction	^33^
	lower or no R amplitude	1/4 (25%)	age-related heart mass loss, old myocardial infarction	^35,36^
	split of P wave	1/4 (25%)	atrial enlargement, dilated cardiomyopathy	^35^
	ST elevation	2/4 (50%)	myocardial infarction	^34^
	Decreased P wave	1/4 (25%)	atrial rhythm	^33^
	at least one EKG phenotype	4/4 (100%)		
i.p., F6 (infectious clone)	no abnormality detected	4/5 (80%)	healthy	
	Irregular heart rate	1/5 (20%)		
i.p., F23 (clinical isolate)	no abnormality detected	4/5 (80%)	healthy	
	Minor arrhythmia	1/5 (20%)		

### Lung infection and inflammation

**Figure 5:**
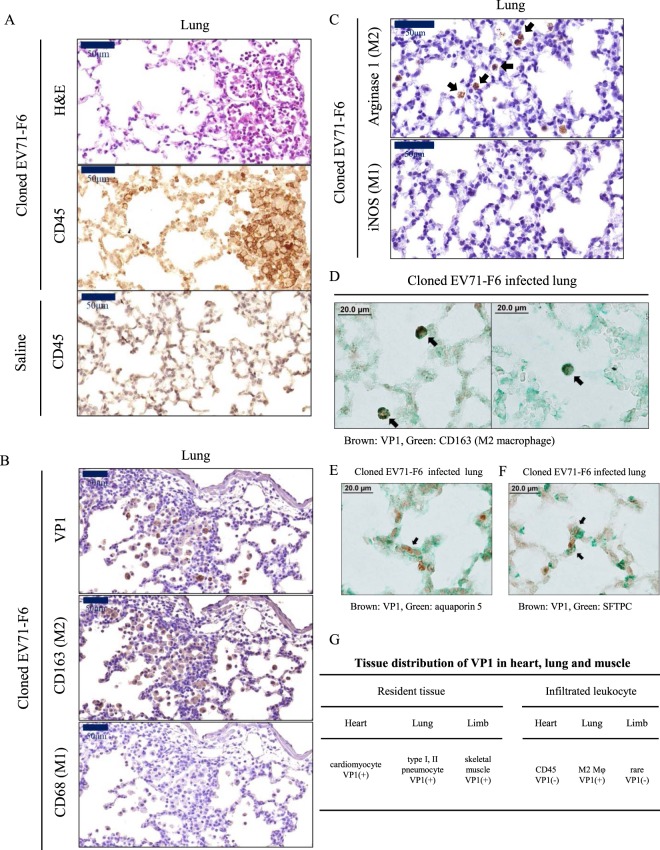
VP1-positive infiltrating M2 macrophage and VP1-positive pneumocytes were detectable in the orally infected lung. Orally infected mice were sacrificed on day 16 post-infection (Fig. 1B). Adjacent lung sections were examined with (**A**) H&E and IHC staining for a pan-leukocyte marker CD45. Massive leukocyte infiltration was detected. (**B**) VP1 and CD163 (M2 macrophage), but not CD68 (M1 macrophage), were detected by IHC in adjacent lung sections. (**C**) Similarly, arginase 1 marker for M2 macrophages, but not iNOS marker for M1 macrophage, was visualized in the alveoli by IHC staining. (**D**) VP1-positive (brown) M2 macrophages (CD163, green) were observed in both panels by IHC double staining. (**E**,**F**) VP1-positive signal was detected in both type I (aquaporin 5) and type II pneumocytes (surfactant protein C). (**G**) Distribution of VP1 in heart, lung and muscle, as well as their respective infiltrating leukocytes.

Heart and lung are two organs closely interacting with each other. Due to the lack of a quantitative and reliable assay method for lung edema in our laboratory, we did not analyze pulmonary edema of infected mice in this current study. Instead, we examined the lung sections by H&E and IHC (Fig. 5A). Massive leukocyte infiltration was visualized by using anti-CD45 antibody. We detected neither VP1 nor infiltrated macrophages in the lung of uninfected control mice (data not shown). In the lung sections from orally infected mice, we could not detect any CD68-positive or iNOS-positive signals for M1 type macrophage (Fig. 5B,C). However, M2 type macrophages in the alveoli were shown to be positive for VP1, CD163, and arginase 1 isoform (Fig. 5B,C). By IHC double staining, we observed colocalization between VP1 and CD163 (Fig. 5D). We did not detect any M2 macrophage in the heart (data not shown). Again, by IHC double staining, cytoplasmic VP1 can be colocalized with membrane-associated aquaporin 5 (type-I pneumocyte marker) (Fig. 5E), and surfactant protein C (type-II pneumocyte marker) within the same cell (Fig. 5F). These results suggest that both types of pneumocytes can be orally infected *in vivo* by EV71. Figure 5G summarizes the tissue distribution of VP1 in muscle, heart and lung, as well as their respective infiltrating leukocytes.

### Sequential expressions of inflammatory cytokines

**Figure 6:**
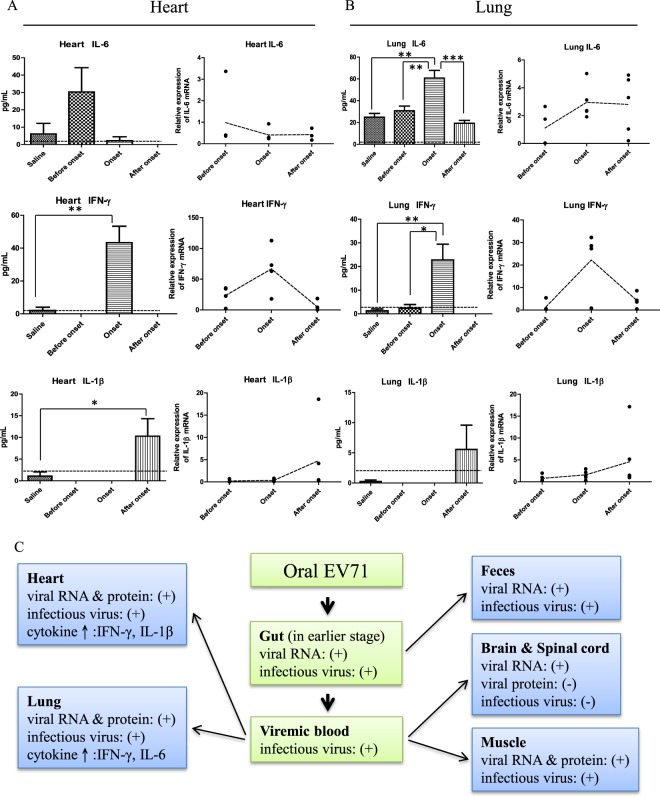
Dissection of virus-host interactions in different tissues in the NOD/SCID mice infected with cloned EV71-F6. (**A,B**) A time course study on the expression profiles of inflammatory cytokines in the cardiopulmonary system by ELISA and RT-qPCR. Experimental design is as outlined in Fig. 1B. Sequential expression of IL6, IFN-γ, and IL-1β were detected in both heart and lung before, during, and after disease onset, respectively. In the ELISA data, dashed lines represent the cutoff value of minimal detectable dose. In the RT-qPCR data, dashed lines represent RNA expression levels relative to the saline control from an averaged value of at least 4 mice, with duplicated samples from each mouse. (**C**) A hypothesis of viral spreading from the oral-gut axis to peripheral tissues via blood circulation at earlier time points post-infection (data from Figs 1, 2, 6). Note: At the moribund stage, no virus was detected in the intestine (Fig. 2A).

In both heart and lung sections, we observed inflammation and leukocyte infiltration by the staining of a pan-leukocyte marker CD45 (Figs 3 and 5A). It is therefore natural to ask whether inflammatory cytokines can be detected in the infected heart and lung (Fig. 6A,B). At the earlier stage before disease onset (8 dpi), IL6 was detectable by ELISA and RT-qPCR in both heart and lung. However, at the disease onset stage (12 dpi), while IL6 peaked in its expression in lung, it could no longer be detected in the heart. In contrast, IFN-γ was detected only at the onset stage in both heart and lung (12 dpi). A lower level of IL-1β was detected only after disease onset in both heart and lung (16 dpi) (Fig. 6A,B). Throughout the entire time course, we detected no TNF-α in heart and lung by ELISA or RT-qPCR.

In summary (Fig. 6C), both cloned virus and clinical isolates of EV71 can target to multiple organs, including the cardiopulmonary system via an oral infection route.

## Discussion

To date, no viral protein has ever been detected in heart and lung from EV71-infected human autopsies. Instead, EV71 protein can be detected in the CNS^2,13,15,17,18^. A generally believed hypothesis is that cardiopulmonary failure in severe patients is indirectly caused by hyperactivated autonomic nerve system in the infected brainstem^13,16,25^. In one report in literature, when NOD/SCID mice were intracerebrally (i.c.) infected with a serially-passaged, mouse-adapted EV71 strain, VP1 protein was detected in the heart. However, i.c. injection into the brain by needles is very different from human natural infection via an oral-fecal route^26^. In another report, ICR mice were orally infected with a mouse-adapted strain (MP4) and VP1 protein was detected in the heart^27^. While these two reports observed VP1 protein in the heart, they are different from our current study in several major ways: (1) Neither reports observed any heart diseases, such as EKG abnormality or apoptosis^26,27^. (2) Neither reports observed any viral protein expression in the lung^26,27^. Similarly, in our own previous studies by i.p.-inoculation with the EV71 clinical isolate, we detected neither VP1 protein nor pathology in the heart and lung^23,28^. (3) Both reports relied on mouse-adapted strains, which deviated significantly from the original parental clinical isolate by accumulating a large number of additional artificial mutations^20^. In fact, using an oral route, the clinical isolate (parental strain 4643) at 10^7^ pfu per mouse failed to infect any ICR mice, while the MP4 mouse-adapted strain infected 70% of ICR mice^27^. In addition to the mouse models, oral infection with EV71 in a gerbil model was reported recently^29^. While heart and lung edema was interpreted from the H&E staining, no viral protein was shown by IHC. The outbred gerbil model is less user-friendly, since no convenient ELISA assay, MHC antibody, or PCR reagents are available for immunopathology studies.

Unexpectedly, in our current study in 3-day-old mice i.p. or orally inoculated with a cloned virus EV71-F6, we detected strong signals of VP1 protein in heart, lung and muscle in nearly 100% of moribund mice (Table 2). This result suggests that the age of the neonatal mice (7-day-old vs. 3-day-old) is a critical determinant for viral tropism to the cardiopulmonary system.

**Table 2:** Tissue tropisms in the NOD/SCID mouse model infected with cloned F6 virus via different routes and host age*.

Inoculation route & age	Heart	Lung	Liver	Muscle	Kidney	Spleen	Brain	Spinal cord	Intestine	average time to death
oral 3-day-old	12/12	11/12	9/9	12/12	3/9	2/9	1/9	0/9	0/9	17–19 dpi
i.p. 3-day-old	3/3	3/3	2/2	2/2	2/2	2/2	N.D.	N.D.	0/2	8–9 dpi
i.p. 7-day-old	0/10	0/10	0/10	10/10	0/10	10/10	0/10	0/10	0/10	9–10 dpi

N.D.: not done.

*Tissue tropism was evaluated by IHC staining for VP1 prorein in tissue sections (Fig. 2).

At present, it remains unclear how EV71 can spread from gut to heart and lung via an oral route. We proposed here that EV71 might initially infect leukocytes and macrophages in the intestine of orally infected mice, and was spread to multiple organs, including muscle, heart and lung by blood or lymphoid circulation (Fig. 6C). This hypothesis is based on the following: (1) Viral RNA and infectious virus (pfu) can be detected in the intestine and feces (Fig. 1H,I). The trend of increasing viral RNA in the time course of infection suggests that EV71 could be proliferating somewhere in the intestine (e.g., Peyer’s patch), despite the lack of detectable VP1 protein signal by IHC in the intestine sections from moribund mice (Fig. 2A). (2) Previously, it was reported that human P-selectin glycoprotein ligand-1 (PSGL1) on the leukocyte membrane could serve as an entry receptor of EV71^30^. EV71 was also shown to *in vitro* infect human PBMC and a monocytic THP cell line^31,32^. (3) Leukocyte infiltration was observed in both heart and lung in the orally infected mice (Figs 3A and 5A). In addition, VP1-positive M2 type macrophages were abundant in lung alveoli (Fig. 5D). Since M2 macrophage is supposed to be more immunosuppressive, they probably played a role in dampening the inflammation in the infected lung. (4) VP1-positive macrophages in the liver (Kupffer cells) were found clustering around the central vein (Fig. 2D). In addition, VP1-positive signals were present in the kidney glomerulus (Fig. 2F), suggesting that EV71 could spread from intestine to liver and kidney through the blood circulation. (5) It is very common to observe muscle infection with EV71 in mouse models^1^. However, myositis in humans has not been reported. In this study, we also detected VP1 and pathology in the hindlimb skeletal muscle (Fig. 2E). It is possible that muscle could serve as an intermediate reservoir of EV71 during the spreading from gut to heart and lung. We observed very abundant amount of VP1, viral RNA, and pfu in hindlimb muscle (Figs 1G and 2E). In the neural spread hypothesis of poliovirus, muscle appeared to serve as a reservoir before invading the CNS^33,34^. (6) We detected viremia in the blood samples at the earlier stage (4/4 at 1 hr post-infection; 8/9 at dpi 1), but diminished EV71 titers at the later stage (3/10 at dpi 3; 0/7 at dpi 5) (Fig. 6C).

Clinically, in 7 out of 7 (100%) autopsied heart samples with left ventricular dysfunction, cardiomyocyte apoptosis was always observed, albeit no viral protein was ever detected in these human heart samples at the autopsy stage^35^. One striking phenomenon in our current oral infection model is the very severe apoptosis in 100% of infected moribund animals (Fig. 3C–G). In contrast to human autopsy, VP1 protein was present in 100% of sectioned heart samples in our mouse model (Fig. 2A). While our finding supports the idea that EV71 can directly attack cardiomyocytes, it is not mutually exclusive with the previous hypothesis based on sympathetic hyperactivity^2,13^. Although we detected viral RNA in CNS throughout the entire time course post-oral infection (Figs 1J,K and 6C), we detected VP1 protein in the brain in one and only one mouse out of a total of 35 mice sacrificed at various stages of disease progression after virus inoculation. It cannot be excluded that most severe patients might have already cleared the virus from their heart and lung before autopsied specimens were collected.

In one report involving EKG analysis of children with enterovirus rhombencephalitis, abnormal EKG phenotypes were observed, including ST depression, prolonged QT interval, and ventricular tachycardia^35^. In our current oral infection mouse model, the most frequent EKG phenotypes include PR segment depression, ST elevation, loss or reduction of R amplitude or P wave and irregular heart rate (Table 1). Overall, these EKG phenotypes observed in our mouse model can all be validated in human heart failure cases in literature^36–41^.

Coxsackie virus family are related to EV71, and known to cause myocarditis^6,42^. In coxsackie virus infection, abnormal EKG patterns can be detected^43–46^. Although some of these EKG abnormalities can be found in infections with either coxsackie virus or EV71, the pattern of a split P wave (Fig. 4B,E) appears to be unique to EV71, since it has not been observed before in the coxsackie virus literature.

Interestingly, coxsackie virus persistent in murine or human hearts was shown to contain a 5′ terminal deletion^47–49^. In our previous^23^ and current studies (Fig. 1), we observed in various tissues significant discrepancy between the amounts of viral RNA (by RT-PCR) and infectious viruses (by pfu). It is possible that in all these cases, defective interfering particles could be generated in the infected hearts.

In contrast to the highly efficient oral infection model, EKG abnormality is very rare in mice i.p.-inoculated at the same age (3-day-old) (Figs 4 vs. S4; Table 1). In the experimental group by the i.p. route, VP1 protein can always be detected by IHC in their hearts and lungs with or without arrhythmia. However, the numbers of apoptotic nuclei in orally infected mice were around 70%, while i.p. infection observed only approximately 20%, and the saline control was around 2% (Fig. 3F). Apoptosis was detected previously in EV71 infected cell culture^50–52^. So far, it has not been reported in literature whether cardiomyocytes can be infected by EV71 *in vitro* or *in vivo*; and if so, whether infected cardiomyocytes can undergo apoptosis. We demonstrated here for the first time that massive apoptosis can be detected in the heart orally infected with EV71 clinical isolates (Fig. 3C–G). Because around 70% of cardiomyocytes are TUNEL-positive (Fig. 3F), and only less than 3% of cardiomyocytes are VP1-positive in the same mouse (Fig. 2B), we attributed the apoptosis mechanism as due to pro-apoptotic cytokines released by the infiltrating leukocytes (Fig. 3A), rather than directly caused by EV71-encoded protein 2B^50^.

Indeed, inflammatory cytokines including IL-6, IFN-γ, and IL-1β, can be detected in both heart and lung by ELISA or RT-qPCR (Fig. 6A,B). Because IL-6 expression in the heart peaked on dpi 8, and declined to undetectable level before disease onset of limb paralysis on dpi 12, it is unlikely that IL-6 is associated with apoptosis detected in moribund mice after disease onset on dpi 16. Expression of STAT-1 and IFN-γ is known to play a critical role in cardiomyocyte apoptosis^53^. Here, in Fig. 6, IFN-γ coincides with disease onset. In this NOD/SCID model without mature T cells, one major source of IFN-γ could be the NK cells^54^. In addition, IL-1β is well known to be associated with the development of heart disease^55–57^. Expression of IL-1β coincides with the cardiomyocyte apoptosis detected at the moribund stage (Figs 3 and 6). It warrants further investigation whether the NLRP3 inflammasome is activated after disease onset in the infected heart^58^. Because we detected no TNF-α nor caspase 1 activation in our orally infected cardiomyocytes at the moribund stage, it is less likely that the apoptosis phenomenon here is related to pyroptosis^59^. Altogether, oral infection with EV71 cloned virus F6 in this neonatal mouse model caused severe cardiomyocyte apoptosis and various EKG abnormalities mimicking human heart diseases. In contrast, i.p.-infected mice died earlier (8 dpi vs. 16 dpi) and presented only mild arrhythmia and moderate apoptosis at the moribund stage. We speculate that the cause of death of i.p.-infected mice is not related to cardiopulmonary collapse. Instead, one plausible cause of death of these i.p.-infected mice could be related to limb paralysis and starvation.

Most studies in this paper were performed using the cloned F6 virus. However, single-strand RNA virus is known to exist in nature as a heterogeneous quasi-species due to the more rapid mutation, recombination, adaptation, selection, and evolution^60,61^. It is therefore a legitimate issue whether all the experimental results here simply reflect an idiosyncracy of the clonal nature of EV71-F6. In Fig. 4, EKG abnormality was always observed in mice infected with either uncloned F23 clinical isolates or F6 cloned virus, despite the fact that the cloned F6 is far less sequence heterogeneous than the F23 clinical isolate (Fig. S5). In our previous oral infection using the clinical isolate F23, we observed a survival rate around 30% using 10^8^ pfu/mouse^23^. Here, we obtained a survival rate around 60% using the cloned EV71-F6 strain at 10^7^ pfu/mouse. Because of the differences in viral strains (F6 vs. F23) and titers (10^7^ vs. 10^8^), we cannot interpret here whether the difference in the survival rates between F6 and F23 is solely due to the difference in sequence divergence or heterogeneity. Further investigation should be warranted to address the significance of EV71 quasispecies in virus spread, virulence, drug resistance and evolution.

In summary, by oral infection with EV71, we detected viral RNA, VP1 protein and plaque forming activity in cardiomyocytes. Such orally infected hearts also exhibited leukocyte infiltration, inflammatory cytokines, severe cardiomyocyte apoptosis, and abnormal EKG patterns. This phenomenon is reminiscent of heart failure in fatal acute human myocarditis^62^.

## Materials and Methods

### Ethics statement

All animal experiments were conducted using protocols approved by Academia. Sinica Institutional Animal Care & Utilization Committee (ASIACUC Protocol number 13-12-622). Research was conducted in compliance with the principles stated in the Guide for the Care and Use of Laboratory Animals, National Research Council, 1996. EV71 clinical isolates^23,28^ were kindly provided by Section of Clincial Virology and Molecular Diagnosis, Department of Laboratory Medicine, Changhua Christian Hospital, Taiwan. Biosafety Committee approval number BSF 005 20080030 from Academia Sinica, Taiwan.

### Construction of the infectious cDNA clone of EV71-F6

A genetic map of the 10.6 kb plasmid pBT-EV71-F6 is as shown in Fig. S1. The full-length EV71-F6 genome (7.4 kb) was constructed by ligation of a 3.1 kb and a 4.7 kb DNA fragments, which were PCR amplified from the EV71-F6 RNA genome. Briefly, the 3.1 kb DNA fragment of the 5′ region of the viral genome was amplified from the cDNA template of the clinical isolate EV71-F6 genome with a forward primer NotI-T7-EV71 (5′-GAGAGCGGCCGC*TAATACGACTCACTATAGGGGG*TTAAAACAGCCTGTGGGTTGCACCCACTCACAGGACCCACG-3′) (underlined NotI cutting site; *italics* indicate T7 promoter sequence) and a reverse primer EV71-R8 (5′-GGACA GCTCCATAT TCA AG-3′). The 3′ region containing approximately 4.7 kb of the EV71-F6 genome was amplified with a forward primer EV71-F7 (5′-GGATATGACATAAC TGG-3′) and a reverse primer NotI-PacI-poly T-EV71 (5′-GAGAGCGGCCGCTTAATTAATTTTTTTTTTTTTTTTTTTTTTTTTGCTATTCTGGTTATAACAAATTTACC-3′) (underline indicates NotI and PacI cutting sites). These two DNA fragments share a common unique ApaLI site at the overlapping region. These two amplified fragments were double digested with NotI and ApaLI, before ligation into the NotI-cleaved 3.2 kb pBT plasmid^63^ (a gift from Dr. Wan-Jr Syu, YMU, Taiwan).

### *In vitro* transcription and RNA transfection

*In vitro* transcription was carried out by T7 *in vitro* Transcription Kit (Ambion, USA) using a template of PacI-linearized DNA of infectious clone EV71-F6. The resultant RNA transcripts were transfected into human rhabdomyosarcoma (RD) cells (ATCC CCL-136) using the Lipofectamine 2000 reagent (Life Technologies, USA). Six days post-transfection, EV71 virus was harvested by three repeat cycles of freeze-and-thaw and stored at −80 °C.

### Cells and virus preparation

EV71 clinical isolates F6 and F23 were kindly provided by Changhua Christian Hospital, Changhua, Taiwan^23^. Both F6 and F23 (genotype B5) were isolated from patients with severe diseases in 2008. The genomic sequences of F6 and F23 strains will soon be submitted for publication in Genbank and elsewhere. Neither isolate contains a G145E mutation in VP1, which was thought to be mouse-adapted^26^. Human RD cells were cultured in Dulbecco’s Modified Eagle medium (DMEM; Gibco) with 10% fetal bovine serum (FBS; Hyclone) and 1% penicillin-streptomycin (Gibco) at 37 °C. For virus preparation, RD cells were cultured in T175 flask with 0.2% FBS, and infected with EV71 at multiplicity of infection (MOI) of 0.01 at 37 °C for 24 hours. Virus was harvested by three cycles of freeze-and-thaw and centrifuged at 3000 × g at 4 °C for 30 minutes. Supernatant was concentrated by ultracentrifugation through a 30% sucrose cushion in Beckman SW28 rotors at 26000 rpm, 4 °C, for 4–6 hours. EV71 pellets were resuspended in phosphate-buffered saline (PBS) for storage at −80 °C, and the viral titer was determined by plaque assay.

### Virus titration

RD cell monolayer was cultured at a density around 5 × 10^5^ cells/well in 6-well plates (SPL life science). EV71 stock was 10-fold serially diluted with DMEM, and RD cells were infected from virus stock at various dilutions. After 1 hour incubation, virus was removed from RD cells, and the cell monolayer was covered with 4 mL DMEM containing 0.3% soft agar (Lonza) and 0.2% FBS at 37 °C. After incubation for 72 hours, RD cells were fixed with 3.7% formalin (Merck) at room temperature for 1 hour, and the number of plaques was scored after crystal violet staining.

### Experimental infection

NOD/SCID mice were purchased from Lasco Co., Ltd. (Taiwan). All mice were housed under specific-pathogen-free conditions in individually ventilated cages. Three-day-old NOD/SCID mice were infected via the oral route with 50 ul of the saline control (phosphate buffered saline), or EV71-F6 (from infectious clone), or EV71-F23 (clinical isolate) at a dose of 10^7^ PFU/50 ul/mouse using a 24-gauge feeding tube. To control for the possible leakage of the inoculum to the respiratory system, we included Trypan Blue in our oral inoculum (Fig. S6). For intraperitoneal infection, 10^7^ PFU of EV71-F6 (from infectious clone) was injected into each 3- or 7-day-old NOD/SCID mouse. The survival rate (around 30%) using 10^8^ pfu/50 ul/mouse in the previous EV71-F23 study^23^ is lower than the survival rate (around 60%) here using the cloned EV71-F6 strain at 10^7^ pfu/mouse. For oral infection studies, moribund mice were usually sacrificed before death on dpi 16. For ip infection, mice reached the moribund stage earlier and were sacrificed around dpi 8. Disease progression in oral infection experiments is divided into three different stages - “before onset” (dpi 8), “onset” (dpi 12–14), and “after onset” (dpi 16).

### Real-time reverse transcription-PCR (RT-PCR)

Total RNAs of tissue homogenates were extracted with a WelPrep cell/tissue RNA kit (Wel-GENE), and were used for reverse transcription by a High-Capacity cDNA reverse transcription kit (Applied Biosystems). The synthetic cDNA was subjected to real-time quantitative PCR (qPCR) analysis by an ABI 7500 system with a Power SYBR green PCR master kit (both from Applied Biosystems). Specific primers for VP1 were CTAGAGGGTACCACCAATCC (forward) and AACCTGGCCAGTAGGAGT (reverse), IL-6 were GATGGATGCTACCAAACTGGAT (forward) and CCAGGTAGCTATGGTACTCCAGA (reverse), IFN-γ were TCAAGTGGCATAGATGTGGAAG (forward) and TGTTGCTGATGGCCTGATTGT (reverse), IL-1β were TGTAATGAAAGACGGCACACC (forward) and TCTTCTTTGGGTATTGCTTGG (reverse), and TNF-α were TTCTCATTCCTGCTTGTGGCA (forward) and TGATGAGAGGGAGGCCATTTG (reverse). The primer sequences of GAPDH (glyceraldehyde-3-phosphate dehydrogenase) were used as the internal control: GTTCCTACCCCCAATGTG (forward) and CAACCTGGTCCTCAGTGTAG (reverse). The amounts of viral RNA were normalized to the levels of GAPDH.

### Virus titer determination

After euthanasia, organs and tissues were harvested, weighed, and homogenized with PBS at 0.1 mg/uL. Viruses in the supernatants of clarified homogenates were first amplified by passaging the tissue-derived virus through the RD cells for one round (24 h) before reinfection with RD cells for the plaque assay. Viral titers were determined by plaque assays and are expressed as plaque forming unit (pfu) per gram.

### Histopathology and immunohistochemistry (IHC)

The euthanized mice were perfused transcardially with PBS, followed by 10% neutral buffered formalin (CHIN I PAO CO., LTD, Taiwan). Tissues were fixed in 10% neutral buffered formalin overnight. Fixed tissues were paraffin embedded, sliced, and stained with H&E by the Pathology Core Laboratory, IBMS, Academia Sinica, Taiwan. Xylene and ethanol were used for deparaffinization and rehydration. For antigen retrieval and enzyme blocking, retrieval buffer pH 6.0 (Dako) and endogenous enzyme blocker (Dako) were used. Slides were washed with PBS, followed by incubation with specific antibodies, including anti-EV71 VP1 (PB7631-D01P, Abnova, Taiwan), anti-CD45 (NB100-77417, Novus, USA), anti-Connexin 43 (ab11370, Abcam, USA), anti-Caspase 3 (cleaved Asp175) (GTX86952, GeneTex, Taiwan), anti-CD68 (ab955, Abcam, USA), anti-CD163 (bs-2527R, Bioss, USA), anti-Arginase 1 (sc-271430, Santa Cruz, USA), and anti-NOS2 (iNOS) (sc-7271, Santa Cruz, USA) antibodies. These slides were washed with PBS and incubated with anti-rabbit, anti-mouse, or anti-rat secondary antibody (from Dako). DAB system (Dako) was used to visualize signals of antigens. Sectioned samples were counterstained with haematoxylin (ScyTek), and mounted with mounting reagent (MUTO Pure Chemicals). For IHC double stain, lung tissue slides were stained by Mouse/Rabbit X Mouse/Rabbit double stain kit (TADS03, BIOTnA Biotech) according to the manufacturer’s instructions using anti-VP1 (PB7631-Do1P, Abnova, Taiwan), anti-CD163 (bs-2527R, Bioss, USA), anti-Aquaporin 5 (GTX11586, GeneTex, Taiwan), and anti-SFTPC (GTX54694, GeneTex, Taiwan) antibody. Images were scanned and presented via Pannoramic 250 FLASH (3DHISTECH Ltd).

### Immunofluorescence assay

For immunofluorescent staining, RD cells were cultured on glass coverslips (18 × 18 mm), and washed with phosphate-buffered saline (PBS) before fixation with 3.7% formalin for 1 hour at room temperature. Fixed cells were washed three times with PBS, and permeabilized with methanol for 10 minutes at room temperature. After the final washing with PBS three times, EV71 protein was stained with a Light Diagnostics^TM^ Enterovirus 71 Monoclonal Antibody (REF3324, Merck). A secondary FITC (fluorescein isothiocyanate) labeled antibody (REF5008, Merck) was then added. After immunostaining, coverslips were mounted on slides in ProLong® Gold antifade reagent with DAPI (4′-6-diamidino-2-phenylindole) (P36931, Invitrogen). Images were collected using a fluorescence microscope.

### TUNEL assay

The heart sections were incubated with TUNEL reagents (Roche) according to the manufacturer’s instructions, and examined by fluorescence microscope. For TUNEL colorimetric assay, heart sections were stained by Click-iT^TM^ TUNEL Colorimetric IHC Detection Kit (C10623, Invitrogen) and FragEL^TM^ DNA Fragmentation Detection Kit, Colorimetric- TdT enzyme (QIA33-1EA, Merck), according to the manufacturer’s instructions. Images were scanned and presented via Pannoramic 250 FLASH (3DHISTECH Ltd). The percentage of apoptosis was estimated quantitatively by scoring the number of TUNEL-positive nuclei divided by the total number of Methyl Green positive signals. A total of 350 Methyl Green positive nuclei were scored for quantitation (50 nuclei/field, a total of 7 fields under microscope).

### Electrocardiography analysis

Uninfected and infected littermates of mice were anesthetized with 1.2–1.5% Isoflurane and placed on a heat pad (TACT-2DF controller, Physitemp.) in supine posture before EKG recording. Body temperature was maintained at constant temperature (35–37 °C) and monitored continuously by a rectal probe. The needle electrodes were inserted subcutaneously in the limbs for lead I EKG recording. The leads were connected to EKG amplifier and acquisition system (Animal Bio Amp., PowerLab 8/30, ADInstruments). Ten minutes later after the insertion of electrodes, EKG data were collected from an episode of 15–20 minutes. The heart rate was maintained at 400 BPM or greater during the entire recording process. A continuous steady 5-min EKG recording was then selected randomly for the waveform and heart rate variant analysis (LabChart, ADInstruments)^64^.

### Cytokine ELISA assay

EV-71 orally-infected mice were sacrificed by CO2. After PBS perfusion, tissue blocs of heart and lung were homogenized with PBS at 0.1 mg/uL. Supernatants were concentrated by centrifugation at 12000 g, 4 °C. The expression levels of IFN-γ (MIF00, R&D systems), IL-1β (MLB00C, R&D systems), IL-6 (M6000B, R&D systems) and TNF-α (MTA00B, R&D systems), was measured according to the vendors’ protocols. To control for the potential perfusion effect on the ELISA cytokine quantitation, qRT-PCR assays for intracellular cytokine mRNAs were performed as described above.

### DNA sequencing

A total of 17 virus isolates from the EV71 F6 infectious cDNA clone and 13 virus isolates from EV71 F23 clinical sample, were plaque purified. Infected RD cells were incubated overnight at 37 °C. Viral RNA was extracted for RT-PCR. The VP1 gene of EV71 was amplified from the cDNA template with a forward primer EV71-F6 (5′-CTACAATCATCTGTCACC-3′) and a reverse primer EV71-R9 (5′-GCTGACTGGATAGTGCTTTC-3′). PCR products of a 1362 bp DNA fragment were sequenced directly for mutation frequency analysis.

### Statistical analysis

Survival rates of infected mice were analyzed with Log-rank test. Clinical scores of experimental mice, cytokine ELISA and TUNEL-positive cardiomyocytes were analyzed by Student’s *t* test. Mutation rates of viral genome were analyzed by Chi-square test. *P < 0.05; **P < 0.001; ***P < 0.0001.

## Supplementary information

Supplementary information
