# Supplementary material for: Enterovirus 71 targets the cardiopulmonary system in a robust oral infection mouse model
Source: Sci Rep. 2019 Jul 31;9:11108. doi: 10.1038/s41598-019-47455-3 (PMC6668393; doi:10.1038/s41598-019-47455-3)
Supplement: Supplementary file 1 — Supplementary information [file 41598_2019_47455_MOESM1_ESM.pdf]

Title: **Enterovirus 71 targets the cardiopulmonary system in a robust oral infection mouse model**

Short title: EV71 oral infection and cardiopulmonary diseases

Authors: [1, 2, 3]Chih-Shin Chang, [2]Chun-Che Liao, [2]An-Ting Liou, [2]Ya-Shu Chang, [2, 4]Ya-Ting Chang, [2, 5]Bing-Hsiean Tzeng, [2]Chien-Chang Chen, and [2]Chiaho Shih*

[1]Program in Molecular Medicine, National Yang-Ming University and Academia Sinica, Taipei, Taiwan

[2] Institute of Biomedical Sciences, Academia Sinica, Taipei, Taiwan

[3]Institute of Microbiology and Immunology, National Yang-Ming University, Taiwan

[4]Taiwan International Graduate Program in Molecular Medicine, National Yang-Ming University and Academia Sinica, Taipei, Taiwan

[5]Cardiovascular Section, Far Eastern Memorial Hospital and Tri-Service General Hospital, National Defense Medical Center, Taipei, Taiwan

* Corresponding author

Mailing address: Institute of Biomedical Sciences, Academia Sinica, Taipei, Taiwan

Tel: 886-2-2652-3996; Fax: 8862-2652-3597; E-mail: cshih@ibms.sinica.edu.tw

**Figure S1.**

A

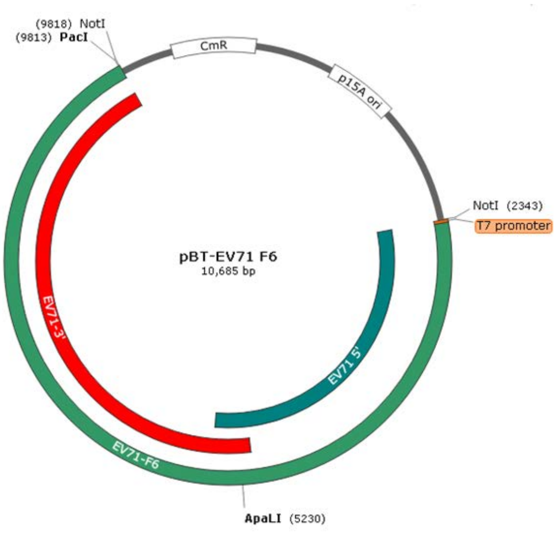


B

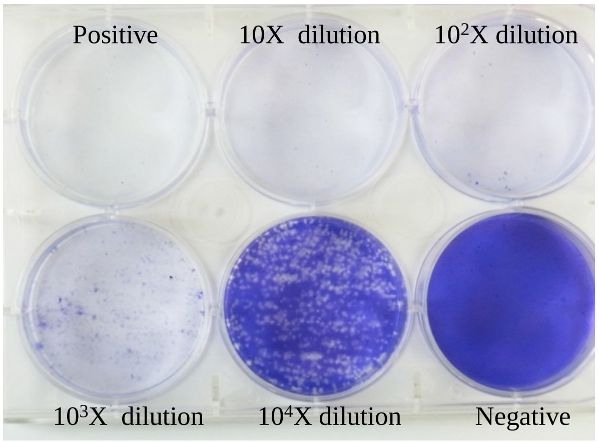


C

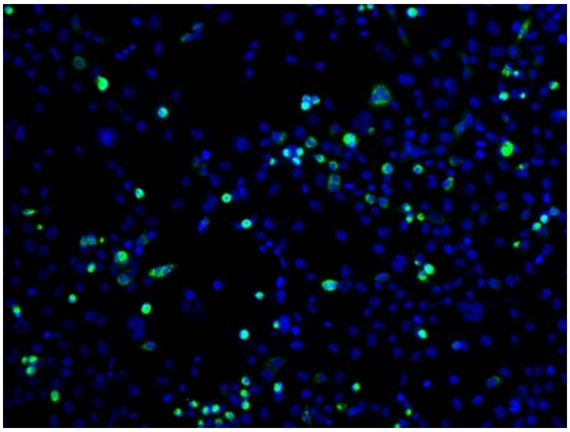

D

i.p. infection of 7-day-old NOD/SCID

| Cloned EV71-F6 (pfu) | Limb paralysis | Death |
|---|---|---|
| $10^5$ | 6/18 (33%) | 6/18 (33%) |
| $10^6$ | 14/31 (45%) | 14/31 (45%) |
| $10^7$ | 22/24 (92%) | 22/24 (92%) |
| $10^8$ | 5/5 (100%) | 5/5 (100%) |

**Figure S1. The EV71-F6 cDNA plasmid cloned from clinical isolates is infectious *in vitro* and *in vivo*.**

Human RD cells were transfected with *in vitro* synthesized EV71 viral RNA, which was generated by *in vitro* transcription using a linearized DNA template from the EV71-F6 plasmid. (A) The EV71-F6 infectious clone was constructed as detailed in Methods. (B) Media were collected from transfected culture on day 6 post-transfection. Plaque assay was conducted to titrate the virus stock in serial dilutions. Infected RD cells were fixed and stained with crystal violet. (C) Viral protein VP1 was visualized in the cloned virus F6 infected RD cells by immunofluorescence microscopy (×200) using an anti-VP1 antibody. (D) Summary of disease manifestations in NOD/SCID mice i.p. infected with the cloned virus F6 at different viral doses ($10^5$ -$10^8$ pfu/mouse).

**Figure S2.**

A

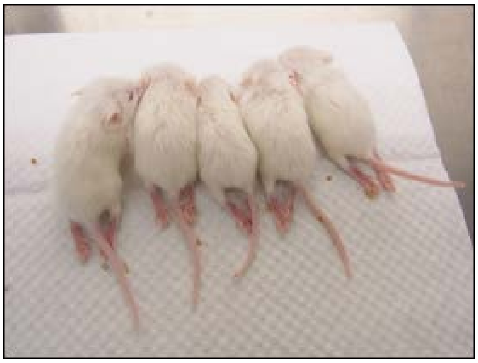

B

Oral infection of 3-day-old NOD/SCID

| Cloned EV71-F6 (pfu/mouse) | Limb paralysis | Death |
|---|---|---|
| $10^6$ | 0/10 (0%) | 0/10 (0%) |
| $10^7$ | 23/43 (53.5%) | 23/43 (53.5%) |

**Figure S2. Oral infection of 3-day-old NOD/SCID mice developed limb paralysis and death.**
The 3-day-old NOD/SCID mice were infected orally with the cloned virus F6 or the saline control (Figure 1A). (A) Orally infected mice developed hind limb paralysis. (B) Paralysis and death were observed in 53.5% of mice orally infected at $10^7$ pfu/mouse.

**Figure S3**.

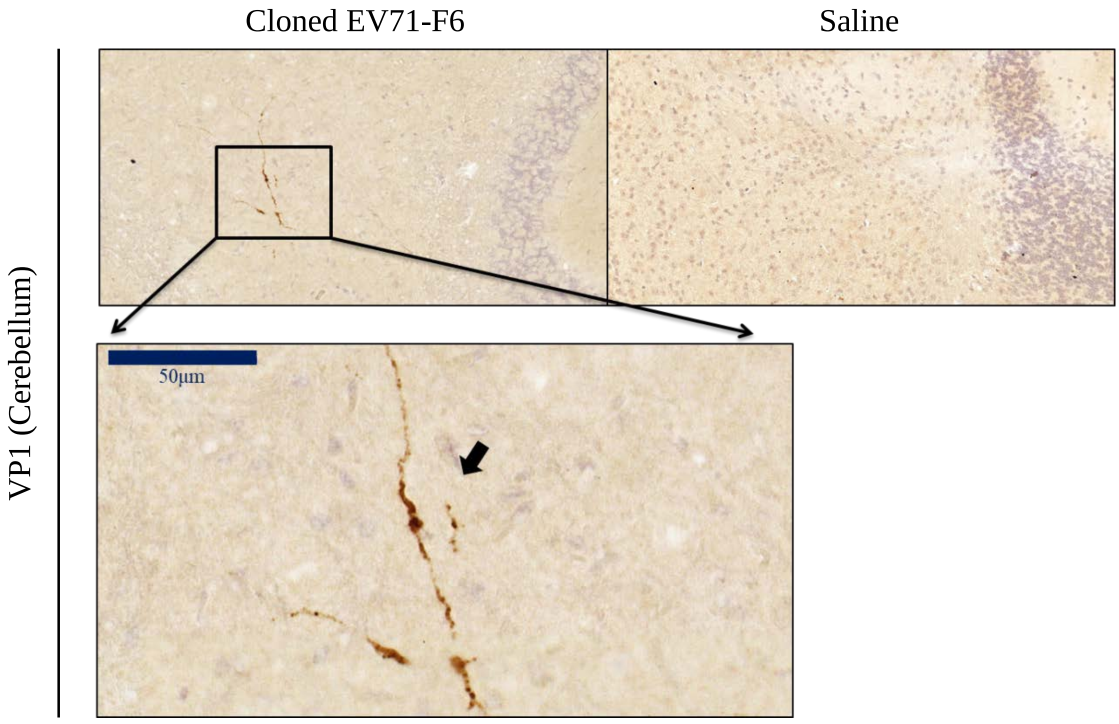


**Figure S3. VP1-positive nerve fibers were detected by IHC in cerebellum in one out of nine mice orally infected with the cloned virus F6.**

**Figure S4.**

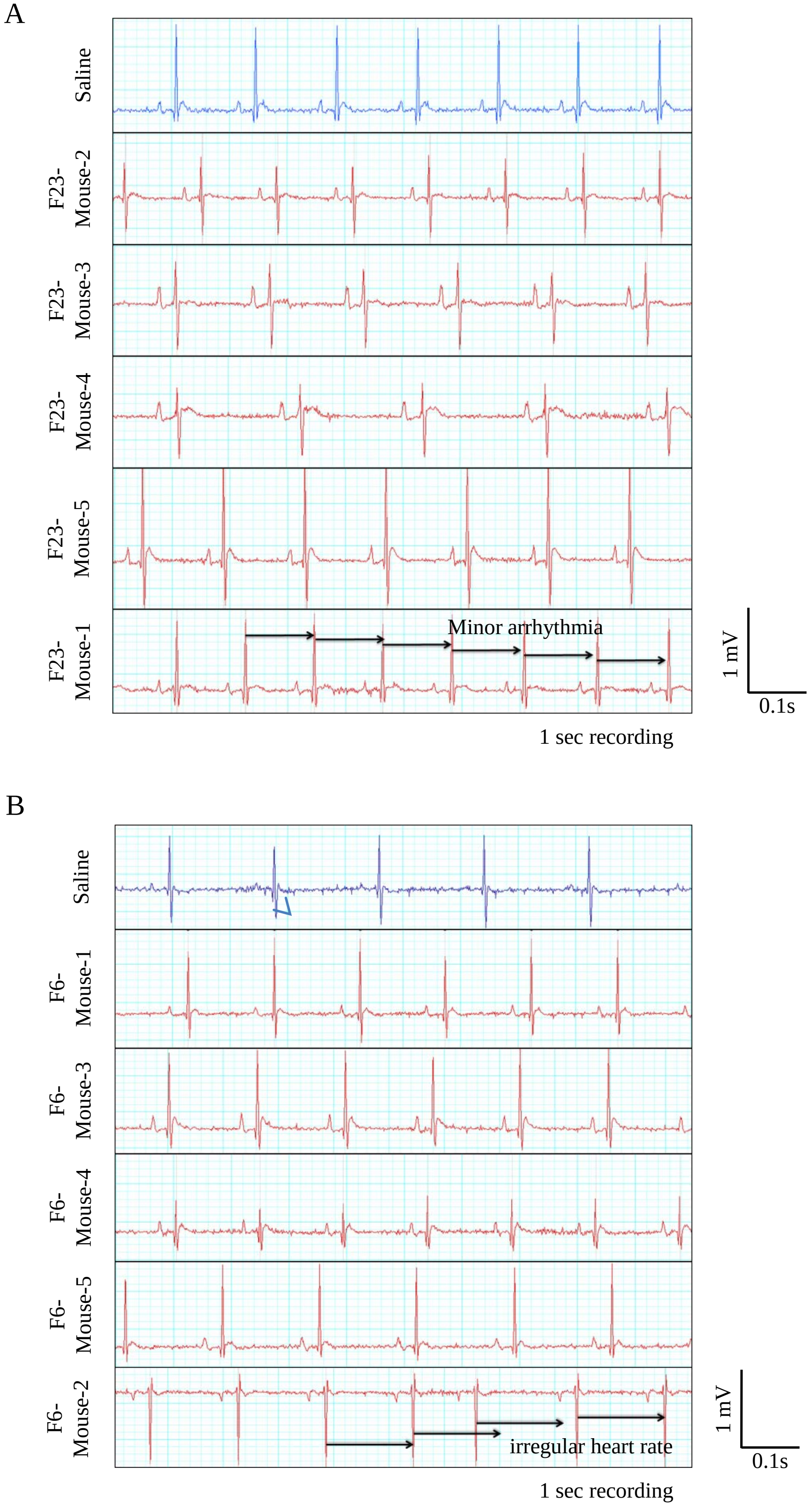


**Figure S4. No apparent EKG phenotype was detected by i.p. inoculation with the EV71 clinical isolate F23 or the cloned virus F6.**

The 3-day-old NOD/SCID mice were i.p. infected with $10^7$ pfu of EV71-F23, EV71-F6 or normal saline. Infected mice were analyzed by EKG on day 10 post-infection. EKG analysis detected only borderline arrhythmia in one out of five mice in (A) F23-Mouse-1 and (B) F6-Mouse-2.

**Figure S5.**

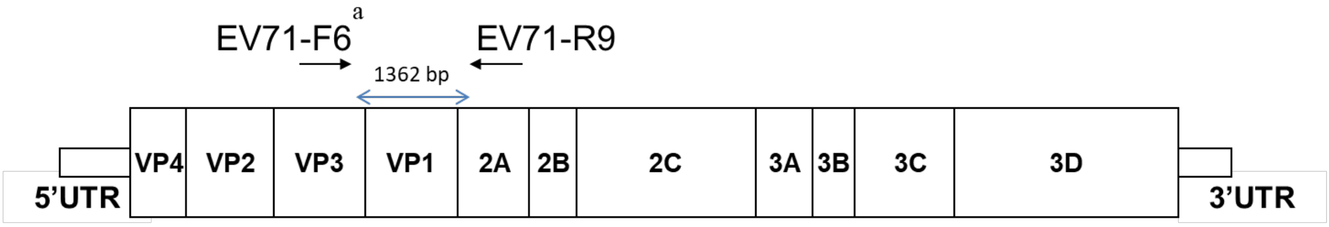


| Virus | Total number of mutations[b] | Mutation rates ($10^{-3}$) |
|---|---|---|
| F6 infectious clone | 35/23154 (1362 X 17) | 1.51 *** |
| F23 clinical isolates | 103/17706 (1362 X 13) | 5.81 |

[a] Primer sequences: EV71-F6 (5'-CTACAATCATCTGTCACC-3') and EV71-R9 (5'-GCTGACTGGATAGTGCTTTC-3').
[b] Mutations observed over the total number of nucleotides sequenced.

* * * $p < 0.0001$.

**Figure S5**. **Genome divergence of F23 clinical isolates is significantly higher than that of the F6 infectious clone.**
DNA sequences of a PCR-amplified DNA fragment of EV71 containing VP1 were compared between 17 independent plaques originated from EV71-F6 infectious cDNA clone. Total number of mutations were calculated by comparing the consensus sequences and those from each individual plaques. Similar analysis was conducted for 13 independent plaques originated from F23 clinical isolates (Materials and Methods). *** $p < 0.0001$ by Chi-square test.

**Figure S6.**

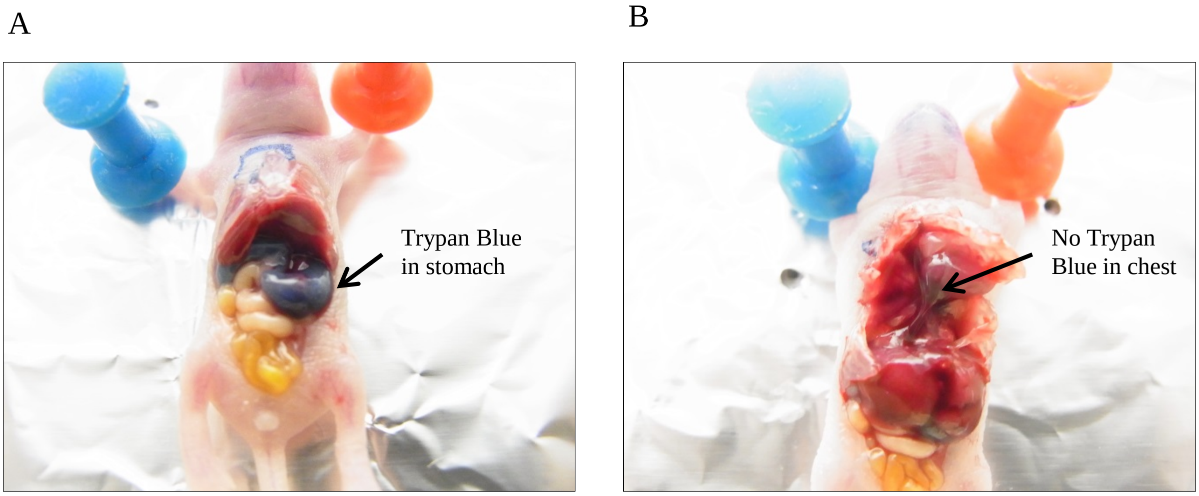


**Figure S6. No leakage of the virus inoculum to the respiratory system as monitored by Trypan Blue indicator**.
(A) Trypan Blue in the stomach was visualized after oral injection into the 3-day-old NOD/SCID mice. (B) No blue color was detected in the chest of mice orally injected with Trypan Blue.

**SUPPLEMENTARY FIGURE LEDENDS**

**Figure S1. The EV71-F6 cDNA plasmid cloned from clinical isolates is infectious *in vitro* and *in vivo*.**

Human RD cells were transfected with *in vitro* synthesized EV71 viral RNA, which was generated by *in vitro* transcription using a linearized DNA template from the EV71-F6 plasmid. (A) The EV71-F6 infectious clone was constructed as detailed in Methods. (B) Media were collected from transfected culture on day 6 post-transfection. Plaque assay was conducted to titrate the virus stock in serial dilutions. Infected RD cells were fixed and stained with crystal violet. (C) Viral protein VP1 was visualized in the cloned virus F6 infected RD cells by immunofluorescence microscopy (×200) using an anti-VP1 antibody. (D) Summary of disease manifestations in NOD/SCID mice i.p. infected with the cloned virus F6 at different viral doses ($10^5$ -$10^8$ pfu/mouse).

**Figure S2. Oral infection of 3-day-old NOD/SCID mice developed limb paralysis and death.**

The 3-day-old NOD/SCID mice were infected orally with the cloned virus F6 or the saline control (Figure 1A). (A) Orally infected mice developed hind limb paralysis. (B) Paralysis and death were observed in 53.5% of mice orally infected at $10^7$ pfu/mouse.

**Figure S3**. **VP1-positive nerve fibers were detected by IHC in cerebellum in one out of nine mice orally infected with the cloned virus F6.**

**Figure S4. No apparent EKG phenotype was detected by i.p. inoculation with the EV71 clinical isolate F23 or the cloned virus F6.**

The 3-day-old NOD/SCID mice were i.p. infected with $10^7$ pfu of EV71-F23, EV71-F6 or normal saline. Infected mice were analyzed by EKG on day 10 post-infection. EKG analysis detected only borderline arrhythmia in one out of five mice in (A) F23-Mouse-1 and (B) F6-Mouse-2.

**Figure S5**. **Genome divergence of F23 clinical isolates is significantly higher than that of the F6 infectious clone.**

DNA sequences of a PCR-amplified DNA fragment of EV71 containing VP1 were compared between 17 independent plaques originated from EV71-F6 infectious cDNA clone. Total number of mutations were calculated by comparing the consensus sequences and those from each individual plaques. Similar analysis was conducted for 13 independent plaques originated from F23 clinical isolates (Materials and Methods). *** $p < 0.0001$ by Chi-square test.

**Figure S6**. **No leakage of the virus inoculum to the respiratory system was monitored by using Trypan Blue as an indicator**.

(A) Trypan Blue in the stomach was visualized after oral injection into the 3-day-old NOD/SCID mice. (B) No blue color was detected in the chest of mice orally injected with Trypan Blue.
